# Case Report: Postacute Rehabilitation of Guillain-Barré Syndrome and Cerebral Vasculitis-Like Pattern Accompanied by SARS-CoV-2 Infection

**DOI:** 10.3389/fneur.2020.602554

**Published:** 2021-01-07

**Authors:** Stefano Colonna, Luciana Sciumé, Federico Giarda, Alessandro Innocenti, Giovanna Beretta, Davide Dalla Costa

**Affiliations:** ^1^Department of Rehabilitation Medicine and Neurorehabilitation, ASST Grande Ospedale Metropolitano Niguarda, Milan, Italy; ^2^Department of Neurology and Stroke Unit, Niguarda Ca' Granda Hospital, Milan, Italy

**Keywords:** SARS-CoV-2, Guillain-Barré syndrome, polyradiculonevritis, cerebral vasculitis, rehabilitation

## Abstract

**Introduction:** The main clinical manifestation of the novel Severe Acute Respiratory Syndrome Coronavirus-2 (SARS-CoV-2) is respiratory issues. Neurological manifestations are being increasingly recognized, including febrile seizures, headache, dizziness, and myalgia, as well as encephalopathy, encephalitis, stroke, and acute peripheral nerve diseases. Cerebral vasculitis is rarely reported. We describe a case of SARS-CoV-2 interstitial pneumonia complicated by flaccid tetraplegia due to Guillain-Barré Syndrome (GBS) associated with a cerebral vasculitis-like pattern.

**Case description:** A 62-year-old man was hospitalized for cough, fever, and severe respiratory failure requiring tracheal intubation and invasive ventilation. The chest Computerized Tomography (CT) showed images related to interstitial pneumonia and the subsequent nasopharyngeal swab confirmed the presence of SARS-CoV-2 infection. During the hospitalization, there was a progressive deterioration of the senses associated with areflexic flaccid tetraplegia. The treatment with high doses of immunoglobulin G (IgG) led to the immediate improvement of the general conditions and a partial response in terms of recovery of the upper limb and of the distal lower limb movements. Subsequently the patient was admitted to our Rehabilitation Unit, where he received an intensive rehabilitation treatment consisting of physiotherapy and occupational therapy. Two months later the patient was discharged at home and able to walk independently even for long distances thanks to the use of Ankle-Foot Orthosis (AFO).

**Conclusion:** In this report, we present the case of a patient with peripheral and central neurological damage occurred later severe pneumonia induced by SARS-CoV-2. The Immunoglobulin G therapy allowed the patient to benefit considerably from early rehabilitation, reaching the walking, increasing the independence in daily living tasks, and enabling safe discharge from hospital to home. Related neurologic complications of SARS-CoV-2 infection suffer a lack of understanding and further investigations should be conducted.

## Introduction

Since the appearance of the first case of coronavirus disease 2019 (COVID-19), the spread of infection has quickly affected millions of people worldwide, and was declared a pandemic by the World Health Organization in March 2020 (1). Severely symptomatic patients may present with pneumonia, acute respiratory distress syndrome (ARDS), acute cardiac dysfunction from myocarditis, and multiorgan failure (2). Although the main clinical presentation is respiratory disease, there is emerging evidence that SARS-CoV-2 infection could be associated with neurological complications, including febrile seizures, headache, dizziness, and myalgia, as well with encephalopathy, encephalitis, stroke, and acute peripheral nerve diseases (3). Cerebral vasculitis is rarely reported (2, 4). features appear to be a combination of nonspecific complications of systemic disease, the effects of direct viral infection, or inflammation of the nervous system and vasculature, which can be para-infectious or post-infectious (4). We aim to report a case of COVID-19 complicated by Guillain-Barré Syndrome (GBS) and central nervous system involvement resembling vasculitis.

## Case Description

**Figure 1:**
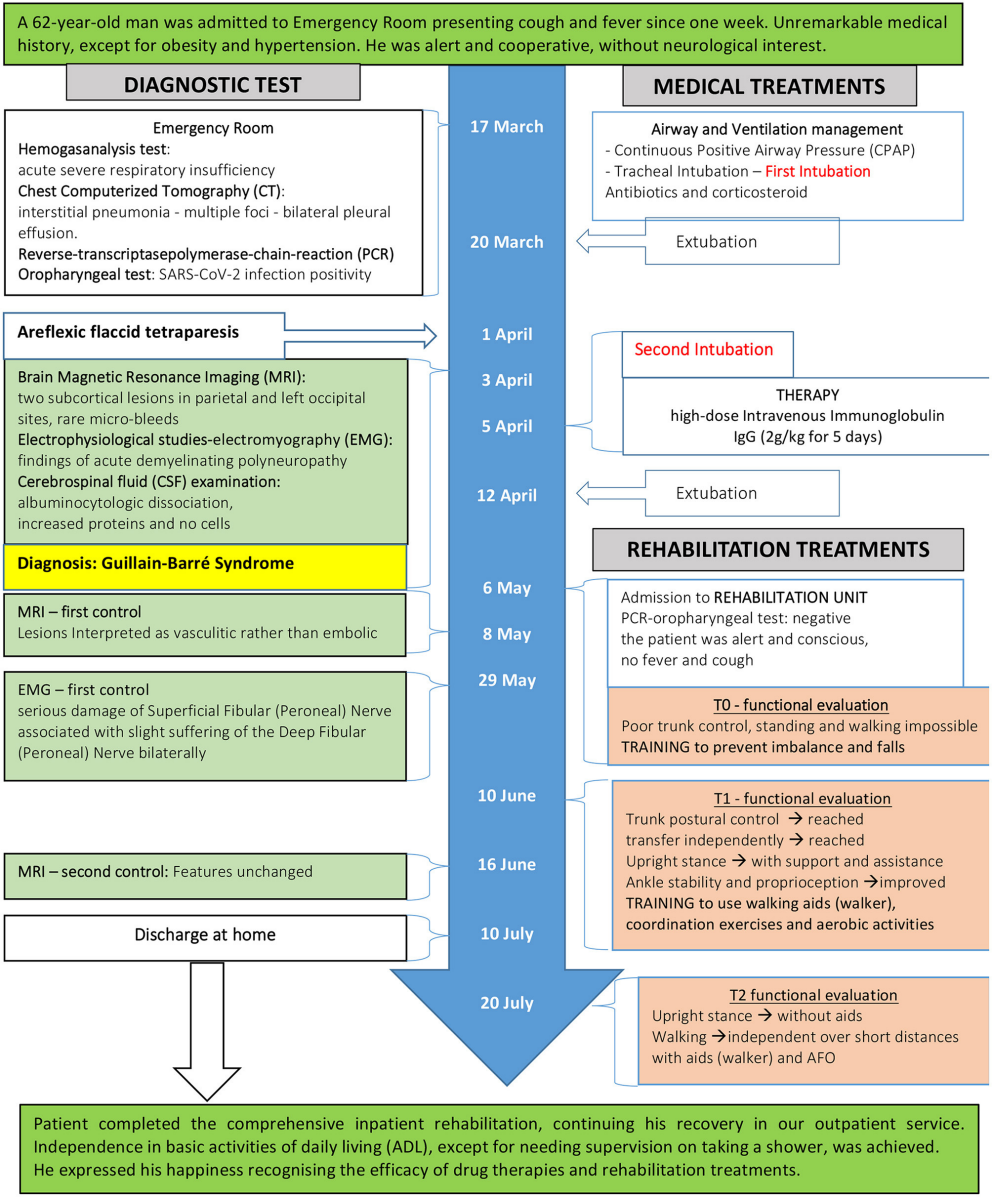
Timeline of symptoms, diagnostic, interventions, and outcomes.

The patient is a 62-year-old man without significant medical history, suffering from high blood pressure and obesity, who was hospitalized between April 2020 and July 2020 in the Neuroscience department of the ASST Grande Ospedale Metropolitano Niguarda ‘Ca Granda in Milan (Italy). On March 17, 2020 the patient was admitted to the Emergency Room of Clinica Polispecialistica in Paderno Dugnano in Italy. He presented with a fever and cough that had been persisting for about a week and that progressively worsened. He was alert and cooperative, without neurological interest. Gastrointestinal symptoms were not recently experienced. At the entrance, hemogasanalysis parameters showed an acute severe respiratory alkalosis: pH: 7.54 – pCO2: 32 mmHg – pO2: 26 mmHg. Chest Computerized Tomography (CT) showed images of interstitial pneumonia with multiple foci in a consolidative evolution and bilateral pleural effusion. Because of this, and considering the clinical suspicion of infection with SARS-CoV-2, a reverse-transcriptase polymerase-chain-reaction (PCR) oropharyngeal swab was performed that confirmed the diagnosis. The patient was transferred to the Intensive Care Unit (ICU) where, at first, he was treated with Continuous Positive Airway Pressure (CPAP), but subsequently intubation was required to improve respiratory gas exchange. He was treated with antibiotic therapy (Piperacillin and Tazobactam 6.75 mg/day for 12 days, Vancomycin 2 g/day for 8 days, Ceftriaxone 2 g/day for 2 days), antiretrovirals (Darunavir/Ritonavir 800/100 mg/day for 12 days), corticosteroid (Methylprednisolone 60 mg for 25 days), and Low-molecular-weight heparin (LMWH – Enoxaparin 2000 IU 2 times for day). Despite concomitant cardiological complications (atrial fibrillation associated with high ventricular response – Heart Rate: 190 beats/min), severe anemia (hemogasanalysis parameters: hemoglobin (Hb): 5.9 g/dl – hematocrit (Ht): 19% on March 30), and acute renal failure (Creatinine level: 4.40 mg/dl – estimated glomerular filtration rate (eGFR): 13.6 mL/min/1.73 m^2^ – blood urea nitrogen level: 192 mg/dl on March 31) improvement of clinical condition and respiratory distress was recorded. On March 20 he was extubated and continued the hospital stay in the General Medicine Department of the same hospital. Figure 1 shows the timeline of symptoms, diagnostic, interventions, and outcomes.

### Diagnostic Assessment and Pharmacological Therapies of Neurological Disorder

Starting from April 1, a progressive worsening of neurological involvement characterized by sensory deterioration associated with flaccid quadriplegia areflexic was detected. For this reason, the patient was transferred to the emergency room of another hospital in Legnano, where more diagnostic exams were performed. The Brain Magnetic Resonance Imaging (MRI) showed two subcortical lesions in the parietal and left occipital sites, with restriction in Diffusion-Weighted Imaging (DWI) and without contrast enhancement. Rare point-like microbleeds without restricted diffusion were also detected in the white matter of both the cerebral lobes, interpreted as suggestive of previous ischemic lesions. Cerebrospinal fluid (CSF) assessment revealed an albuminocytologic dissociation with increased glucose (166 mg/dl, normal 45–80 mg/dl), protein level (51 mg/dl, normal 8–43 mg/dl), and no cells. SARS-Cov-2 RNA was not tested in CSF. Electrophysiological studies were performed: the common peroneal nerve showed no excitation on the left and marked lower amplitude of nerve conduction on the right, as well as a conduction block at the popliteal fossa. Significantly prolonged distal motor latencies and temporal dispersion of the compound muscle action potentials (CMAP) at four limbs muscles, absent F-waves, and reduced motor nerve conduction of the tibial, median, and ulnar nerves on both sides were recorded, as well as slightly reduced sensory potential amplitude size. Sensory conduction pathways of the median, ulnar, and sural nerves were normal. Electromyography (EMG) needle electrode showed no denervation signs. Motor unit recruitment was not assessable. The findings described were consistent with the diagnosis of acute inflammatory polyradiculoneuritis. On the basis of these results, the diagnosis of Guillain Barré Syndrome (GBS; Acute Inflammatory Demyelinating Polyneuropathy or AIDP variant) was done. In this context, we hypothesized that AIDP could be the result of an autoimmune reaction in the course of infection with SARS-CoV-2 (5, 6). Once more the patient was intubated then treated with high-dose Intravenous Immunoglobulin (2 g/kg from April 5 to April 10). GBS prognosis can be estimated by applying the modified Erasmus Guillain-Barré Syndrome outcome score (mEGOS) (7, 8). The results were 8/9 at admission and 11/12 at day 7 of hospitalization, pointing to a poor outcome. Negative mood and sometimes depression were reported mainly due to loss of autonomy and complete dependence on ADLs. On April 12, after extubation, the hospital stay continued in the General Medicine Department, where he underwent hemodialysis for acute renal failure on April 15 (Creatinine level 5.50 mg/dL and diuresis of 500 ml in 24 h). On May 6, general health condition was stable. The muscle strength enhanced, although weakness of the proximal upper limb and of the distal lower limb remained relevant. Consequently, he was admitted to our Rehabilitation Unit (RU) to continue the recovery.

### Rehabilitation Assessments and Treatments

At admission the patient was alert and conscious, body temperature was 37.2°C, there was no cough, and the last PCR-oropharyngeal test was negative. Vital signs' measurements were: blood pressure: 125/70 mm Hg, heart rate: 74 beats/min, and oxygen saturation: 95% on room air. Standard laboratory tests detected white blood cells count (17.600), Hb (8 g/dl), c-reactive protein (6.5 mg/dl), creatinine level (4.8 mg/dl), and blood urea nitrogen level (117 mg/dl). Erythropoietin was prescribed to treat anemia. No electrocardiographic changes were visible. At the neurological examination cranial nerves were intact and no speech disorders or swallowing problems were noticeable. All sensations were preserved. Motor clinical assessment was characterized by a marked loss of muscle mass and tetraparesis, evident in the proximal upper and lower limbs (strength muscle was of grade 3/5 at deltoid, biceps, extensor carpi radialis, iliopsoas, and quadriceps) and more intense at ankle dorsiflexor muscles (grade 0/5 at anterior tibial muscles), as measured by the British Medical Research Council muscle strength grading system (9). Deep tendon reflexes were absent; muscle tone was normal in four limbs. Depressive symptoms described during ICU stay were reduced: drug therapy (Citalopram, 20 mg/day), psychological support, and contacts with family members, once a day because of COVID-19 restrictions, gradually had a positive effect on emotions. In order to monitor rehabilitation effectiveness and outcomes, the staff applied the following measurements: Modified Rankin Scale (MRS) (10), Modified Barthel Index (MBI) (11), Trunk Control Test (TCT) (12), Short Physical Performance Battery (SPPB) (13), Berg Balance Scale (BBS) (14), Time-Up and Go (TUG) (15), and 6 Min Walking Test (6MWT) (16). The first evaluation was performed on May 7 (T0) and the final one on July 20 (T2). An intermediate assessment was carried out on June 10 (T1) when it was possible to administer all the tests. The early rehabilitation program consisted of, twice a day, a 45 min' physiotherapy (PT) session alternated with 30 min' occupational therapy (OT) for 6 days a week. At the first stage, the trunk control was poor in a sitting position and the patient needed assistance in transfers, thus a standing position and walking were not possible. Rehabilitation programs were initially aimed to prevent deconditioning and development of skin ulcers, as a result of bed rest and physical inactivity, and muscle shortening and joint contracture, as consequence of motor weakness. Proper bed positioning with frequent postural changes, and sitting posture on the bed through back support and on a wheelchair were provided. Time to sit was gradually increased up to 4 h per day after a week. To reduce fatigue, pulmonary rehabilitation included breathing control, chest-abdominal coordination exercises to stimulate a proper recruitment of the diaphragm muscle, and positive expiratory pressure (PEP) bottle exercise to increase the pulmonary volume. To improve muscle mass and power, initial exercises included gentle strengthening involving isometric manual-resistive exercises, followed by upper and lower limb active exercises and manual progressive resistive mobilization, carefully tailored to the clinical condition of the patient. After 10 days, trunk postural control was achieved and a few days later the ability to transfer independently was also gained. When the upright stance was reached with support and assistance of the PT/OT, more specific training was set up to prevent imbalance and falls. This training involved balance in static and dynamic conditions and active exercises of lower limb and trunk muscles. Due to impaired ankle stability, great care to proprioception (sense of body position and movement) was taken (T1 – 1 month later). In the last month (T2 – 2 months later) the rehabilitation program focused on walking recovery, at the beginning over short distances with supports, assistance, and Ankle-Foot Orthoses (AFO) to contrast bilateral foot drop, then by learning to use walking aids (walker). Coordination exercises and aerobic activities were involved to reach the best performance.

### Diagnostic Follow-Up

**Figure 2:**
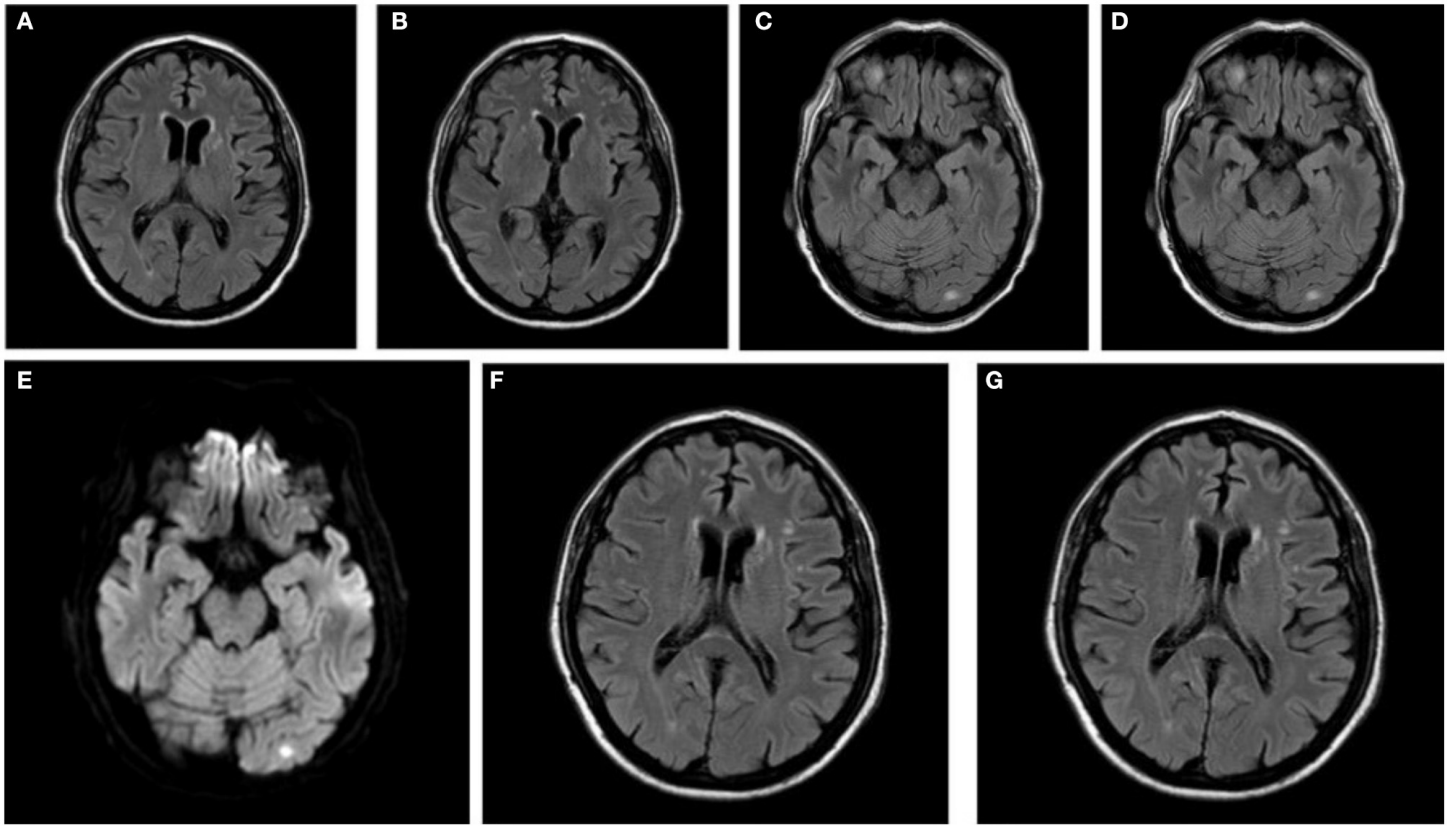
Shoot from brain MRI of May 8 in (**A–E): (A,B)** show T2-FLAIR acquisition. **(C)** Shows occipital lesion in T2-FLAIR acquisition. **(D)** Shows parietal lesion in T2-FLAIR acquisition. **(E)** Shows occipital lesion positive in DWI. Shoot from Brain MRI of June 16: **(F,G)** show T2-FLAIR acquisition.

A first control MRI was checked on May 8 (Figures 2A–E). The exam displayed small T2 fluid-attenuated inversion recovery (FLAIR) focal hyperintensities in the subcortical white matter with restricted diffusivity in the left occipital and parietal cortex (IMG); due to the size and location, the multiple lesions were interpreted as vasculitic rather than embolic. On June 16, the neuroimaging features between the first and second MRI were unchanged, showing ischemic lesions in the left parietal and occipital lobes without restricted diffusion and still suggestive for vasculitic-like lesions (Figures 2F,G). The EMG of May 29, additionally, corroborated the diagnosis of GBS, defined by serious damage of the Superficial Fibular (Peroneal) Nerve associated with slight bilateral suffering of the Deep Fibular (Peroneal) Nerve (Table 1).

**Table 1:** Summary table of results of electromyographic examination of May 29.

	**Spontaneous**					**MUAP**			**Recruitment**
**Muscle**	**IA**	**Fib**.	**PSW**	**Fasc**.	**H.F**.	**Amp**.	**Dur**.	**PPP**	**Pattern**
L. Tib. Anterior	N	3+	3+	None	None	–	–	–	Absent
L. Ext. Dig. Brevis	N	3+	3+	None	None	–	–	–	Absent
R. Ext. Dig. Brevis	N	3+	3+	None	None	N	N	N	Absent
L. Gastrocn (Med)	N	None	None	None	None	2+	1+	2+	Reduced
R. Gastrocn (Med)	N	None	None	None	None	2+	1+	2+	Reduced
L. Vast. Lateralis	N	None	None	None	None	N	N	N	Sub Interference
R. Vast. Lateralis	N	None	None	None	None	N	N	N	Sub Interference

*Abbreviation of Muscles. L. Tib Anterior, Left Tibialis Anterior muscle; L. Ext. Dig. Brevis, Left Extensor Digitorum Brevis muscle; R. Ext. Dig. Brevis, Right Extensor Digitorum Brevis muscle; L. Gastrocn (Med), Left Gastrocnemius muscle medial; R. Gastrocn (Med), Right Gastrocnemius muscle medial; L. Vast. Lateralis, Left Vastus Lateralis muscle; R. Vast. Lateralis, Right Vastus Lateralis muscle. Abbreviation of examination. Spontaneous IA; Spontaneous Fib., Fibrillation; Spontaneous PSW, PolySpike Waves; Spontaneous Fasc., Fasciculations; Spontaneous H.F., High Frequency; MUAP, Motor Unit Action Potential; Amp., Amplitude; Dur., Duration; PPP, PolyPhasic Potential - Recruitment Pattern. Abbreviation Results: N, Normal*.

### Outcomes of Rehabilitation

The rehabilitation process promoted a gradual increase of strength muscle and led to functional recovery. Assessment results are presented in Table 2. Walking was initially possible with the support of a four-wheeled walker for medium distances (6MWT: 237 meters) with walking speed of 2.37 Km/h, using two Ankle-Foot Orthoses (AFO), as a consequence of bilateral weakness in the ankle dorsiflexion and plantarflexion. At discharge, 75 days after admittance to our RU, the patient achieved the restoration of strength performance at proximal limb muscles (grade 5/5), however muscle weakness of ankle and toes dorsiflexion persisted (grade 3/5 on the right; grade 1/5 on the left). Balance control was upgraded (BBS: 50/56), as well as walking technique and aerobic endurance. Wearing AFOs, but without assistance or aid, he walked for long distances (6MWT: 345 meters) with a speed of 3.45 Km/h. Independence in basic activities of daily living (ADLs), except for needing supervision on taking a shower, was achieved. After completing the comprehensive inpatient rehabilitation, he continued his recovery in our outpatient service.

**Table 2:** Results of motor and functional assessment.

	**MRS** **(points)**	**MBI** **(points)**	**TCT** **(points)**	**SPPB** **(points)**	**BBS** **(points)**	**TUG** **(seconds)**	**6MWT** **(meters)**
**TIMING**							
**T0 (**May 7, 2020)	5/5	19/100	36/100	0/12	8/56	N.E.	N.E.
**T1** (June 10, 2020) (4WW + AFO)	3/5	46/100	100/100	3/12	31/56	22	237
**T2** (July 20, 2020) (AFO)	1/5	100/100	100/100	12/12	50/56	10	345
**RELIABLE CHANGE**							
T0–T1	N.E.	SEM = 1.45 (17) RC = 18.05	N.E.	SEM = 1.42 (18) RC = 5.53	SEM = 2.93 (19) RC = 5.56	N.E.	N.E.
T1–T2	N.E.	SEM = 1.45 RC = 31.22	N.E.	SEM = 1.42 RC = 4.48	SEM = 2.26 RC = 5.94	SEM = 1.14 (20) RC = 7.45	SEM = 22 (18) RC = 3.47

*Abbreviations Tests: MRS, Modified Rankin Scale; MBI, Modified Barthel Index; TCT, Trunk Control Test; SPPB, Short Physical Performance Battery SPPB; BBS, Berg Balance Scale; TUG, Time-Up and Go; 6MWT, 6 Minutes Walking Test. Abbreviations Aids: 4WW, four-wheeled walker; AFO, Ankle-Foot Orthoses. Abbreviation Result: N. E., Not Executable. Abbreviation Statistic: RC, Reliable Change method calculated as RC = xt1-xt2/Sdiff; Sdiff = √2(SEM^2^) - SEM, standard error measurement. The value is attributed considering: SPPB (Older Adults), 6MWT and MBI (Stroke), BBS (Stroke; T0–T1 individuals who ambulate with assistance; T1–T2 individuals who ambulate independently)*.

## Discussion

The respiratory system is the most commonly affected by SARS-CoV-2, but other organ manifestations have been described involving the heart, kidney, and gastrointestinal system. Previous data indicate that the virus is capable of causing an excessive immune reaction with an increased level of cytokines, such as Interleukin-6 (IL-6) (5, 21). It seems that these immunological processes stimulate an inflammatory cascade, leading to extensive tissue damage, including of the nervous system, with variable clinical implications (3). To date, there is no evidence that SARS-CoV-2 is highly neurovirulent (4), though neurological signs, such as nausea, vomiting, myalgia, dizziness (6), hypogeusia, hyposmia, and impaired consciousness (22), were observed as first symptoms. According to other findings, the body's innate and adaptive immune responses to infection as well as the virus itself could be responsible for both central and peripheral neurological damage (4). With regard to the peripheral nervous system (PNS), GBS is an immune-mediated disease; although mechanisms for coronavirus PNS disease are not well understood, viral infection is likely to cause an immune response and a pro-inflammatory state that results in dysimmune disorders including GBS. GBS associated with SARS-CoV-2 might follow the pattern of a para-infectious mechanism, instead of the classic post-infectious profile, as reported in GBS associated with the Zika virus (23). The mechanisms of CNS vascular disease related to coronavirus are probably more complex and multifactorial. The main mechanism is linked to a pro-inflammatory state with consequent activation of thrombotic pathways and microvascular damage. Stroke can result from other mechanisms: an acute infection can trigger atrial fibrillation or endothelial dysfunction can lead to vascular complications. An involvement of brain parenchyma by the virus is also possible. Finally, similarly to what happens to other viruses (e.g., varicella zoster), immune response and pro-inflammatory status related to coronavirus-types can result in a vasculitic process (4). Regarding vasculitis lesions, histologic evidence has been reported in many organs such as the lung, liver, kidney, or skin of patients with COVID-19 (24–26), but cerebral vessels have not yet been investigated. To our knowledge, a single case report about COVID-19 and complications with a CNS vasculitis-like pattern was published, showing extensive cerebral small-vessel ischemic lesions resembling cerebral vasculitis (2). On the other hand, it is known that the prevalence and degree of cerebral white matter lesions increased with age (27). What we observed in our case is that brain lesions might have a different pathogenesis. The cerebral ischemic lesions were acute (restricted diffusion in MRI), multiple, and in different vascular territories and in cortical locations; considering the number of acute ischemia it is unlikely that they could originate from an atheromatous mechanism while, conversely, the small size makes an embolic cause unlikely. It might be considered that the pattern may be suggestive of a vasculitic origin: considering the association with both another dysimmune disease and SARS-CoV-2, we could speculate that the viral infection could have caused a dysimmune-response involving the nervous system, as described below. Diagnosis of SARS-CoV-2 infection of our patient was made at admission to the hospital and, primarily, he was treated with respiratory support and drugs, as referenced above. Neurological involvement appeared 14 days later and it was revealed by an acute and severe onset with cognitive impairment and flaccid, areflexic quadriplegia. Considering the temporal association, we can conjecture that SARS-CoV-2 may have contributed to the development of GBS in this patient. One article reported the interval of 5–10 days between the onset of viral illness and the first symptoms of GBS for five patients (28). This time is similar to the interval seen with GBS that occurs during or after other infections (29). Many case report series described increased GBS incidences (up to more than 5 times higher than expected) in COVID-19 affected areas (28). However, some limitations are worth noting. This syndrome was difficult to explore, probably because of the rarity of clinical manifestations. Furthermore, the alternative explanation that the patient coincidentally developed GBS of an unknown cause should be considered. The assumption is that the viral infection might have caused a dysimmune response involving both the PNS and CNS. Indeed, CNS involvement may also be determined by a dysimmune mechanism, with ischemic lesions of possible vasculitic origin, with a monophasic course. Excellent response to Intravenous Immunoglobulin (IgG) is consistent, although not conclusive with the hypothesis. A quick recovery of clinical status was stimulated with early rehabilitation. We applied several measurements for different skills. More specifically, MRS was used to evaluate general motor improvement; TCT, SPPB, and BBS was used for balance ability. TUG and 6MWT was used to assess walking speed, MBI was used for independence in ADLs. In particular, we observed a progressive strength recovery, primarily in upper limbs, hand grip, and manual skills. Upright position was restored in about 30 days. As expected, the clinically significant improvement of lower-limb muscle strength, as well as walking ability, occurred during the rehabilitation time. At the same time, this better clinical status led to significant improvements in mood and well-being, as well as a significant reduction in anxiety.

Because measuring change in outcome evaluations in an individual can be used to address both statistical and clinical significance, we applied the Reliable Change (RC) index, proposed by Jacobson and Truax in 1991 (30, 31). RC index indicates whether an individual change score is statistically significantly greater than a difference that could have occurred due to random measurement error alone (32). It is computed by dividing the difference between the pre-treatment (Xt1) and post-treatment (Xt2) scores by the standard error of the difference (Sdiff) between the two scores.

$$\begin{array}{l} RC=xt1-xt2/Sdiff\;Sdiff=\surd 2(SEM^{2}) \end{array}$$

If the RC is >1.96, then the difference is reliable; a change of that magnitude would not be expected due to the unreliability of the measure? Conversely, if the RC score is 1.96 or less then the change is not considered to be reliable, it could have occurred just due to the unreliability of the measurement. RC index requires knowledge of distribution scores for the normal and disordered populations under scrutiny (33, 34), and, unfortunately, in the case of GBS these data are not known. Nonetheless, considering similar populations affected by neurological disease (e.g., stroke), the standard error measurement (SEM) is calculated for some of the scales we administered. As shown in Table 2, RC was calculated for 6MWT, TUG, BBS, SPPB, and MBI. These results are clinically and statistically significant. For TCT and MRS a 30% change from baseline might be considered as a clinically meaningful improvement for individual patients, comparing measurements at different follow-ups (35).

An additional consideration is about walking speed at discharge (3.45 Km/h). It was higher compared to one other reported by Novak et al. (36) in GBS (2.8 km/h), but lower considering the normal walking speed for men aged between 60 and 69 years (4.82–5.16 Km/h) (37).

In summary, in this case report we have reported the clinical history of a patient who suffered from peripheral (GBS) and central (vasculitis) neurological involvement at one time, and then later from SARS-CoV-2 infection. We have only hypothesized a possible association between infection and neurological symptoms. Further studies should be conducted to support a causal relationship and better understand this possible link. Quick detection of neurological symptoms and diagnosis are fundamental to set up the appropriate therapy. IgG infusion allowed the patient to benefit considerably from early rehabilitation, achieving walking, increasing independence in daily living tasks, and enabling safe discharge from hospital to home. The patient was fully aware of the seriousness of his illnesses, especially in the early stages, characterized by a long time spent in the ICU due to severe pneumonia then was made even worse by the tetraplegia. Loss of autonomy (poorly tolerated by the patient) and removal from the family, necessary due to the infectious state, contributed to the worsening of the mood. Muscle strength recovery and consequent autonomy in ADLs gave the patient a positive emotional boost, strengthened by reuniting with relatives after COVID-19 restrictions imposed during hospitalization in the Rehabilitation Unit relaxed. At discharge he expressed his happiness recognizing the efficacy of drug therapies and rehabilitation treatments.

## Data Availability Statement

The original contributions presented in the study are included in the article/Supplementary Materials, further inquiries can be directed to the corresponding author/s.

## Ethics Statement

Ethical review and approval was not required for the study on human participants in accordance with the local legislation and institutional requirements. The patients/participants provided their written informed consent to participate in this study. Written informed consent was obtained from the participant for the publication of any potentially identifiable images or data included in this article.

## Author Contributions

SC wrote the first draft, was responsible for data collection, performed data interpretation. LS performed the literature search and revised data interpretation. FG performed data interpretation and the literature search. AI prepared Figure 2, performed data analysis, and interpretation. GB revised data interpretation. DD conceived the study, prepared Figure 1/Supplementary Material, performed statistical analysis, revised data interpretation, and the final manuscript. All authors contributed to the article and approved the submitted version.

## Conflict of Interest

The authors declare that the research was conducted in the absence of any commercial or financial relationships that could be construed as a potential conflict of interest.
